# Supplementary material for: Association of HDL Subfraction Profile with the Progression of Insulin Resistance
Source: Int J Mol Sci. 2023 Sep 1;24(17):13563. doi: 10.3390/ijms241713563 (PMC10488248; doi:10.3390/ijms241713563)
Supplement: Supplementary file 1 [file ijms-24-13563-s001.zip › ijms-2558755-supplementary.pdf]

Supplementary Table S1. Nomenclature and physical characteristics of high-density lipoprotein (HDL) cholesterol subfractions according to the separation technique used.

	Very large HDL (HDL-VL)	Large HDL (HDL-L)	Medium (or intermediate) HDL (HDL-M or HDL-I)	Small HDL (HDL-S)	Very small HDL (HDL-VS)
Density range, g/mL	1.063 – 1.087	1.088 – 1.110	1.110 – 1.129	1.129 – 1.154	1.154 – 1.210
Size range, nm	12.9 – 9.7	9.7 – 8.8	8.8 – 8.2	8.2 – 7.8	7.8 – 7.2
Density gradient ultracentrifugation	HDL$_{2b}$	HDL$_{2a}$	HDL$_{3a}$	HDL$_{3b}$	HDL$_{3c}$
Density range, g/mL	1.063 – 1.087	1.088 – 1.110	1.110 – 1.129	1.129 – 1.154	1.154 – 1.210
Gradient gel electrophoresis	HDL$_{2b}$	HDL$_{2a}$	HDL$_{3a}$	HDL$_{3b}$	HDL$_{3c}$
Size range, nm	12.9 – 9.7	9.7 – 8.8	8.8 – 8.2	8.2 – 7.8	7.8 – 7.2
2-D gel electrophoresis	α-1	α-2	α-3	α-4	α-5
Size range, nm	11.2 – 10.8	9.4 – 9.0	8.5 – 7.5	7.5 – 7.0	6.0 – 5.0
Nuclear magnetic resonance (NMR)	Large HDL-P	Medium HDL-P		Small HDL-P	
Size range, nm	12.9 – 9.7	9.7 – 8.8	8.8 – 8.2	8.2 – 7.8	7.8 – 7.2
Ion mobility	HDL$_{2b}$	HDL$_{2a}$ and HDL$_{3}$			
Size range, nm	14.5 – 10.5	10.5 – 7.65			
Lipoprint HDL system	HDL-L:<br>HDL-1, -2, -3		HDL-I:<br>HDL-4, -5, -6, -7	HDL-S:<br>HDL-8, -9, -10	

nm: nanometer, HDL-P: HDL particle
